# From early stress to 12-month development in very preterm infants: Preliminary findings on epigenetic mechanisms and brain growth

**DOI:** 10.1371/journal.pone.0190602

**Published:** 2018-01-05

**Authors:** Monica Fumagalli, Livio Provenzi, Pietro De Carli, Francesca Dessimone, Ida Sirgiovanni, Roberto Giorda, Claudia Cinnante, Letizia Squarcina, Uberto Pozzoli, Fabio Triulzi, Paolo Brambilla, Renato Borgatti, Fabio Mosca, Rosario Montirosso

**Affiliations:** 1 NICU, Department of Clinical Sciences and Community Health, Università degli Studi di Milano, Fondazione IRCCS Ca' Granda Ospedale Maggiore Policlinico, Milano, Italy; 2 0–3 Centre for the at-Risk Infant, Scientific Institute, IRCCS Eugenio Medea, Bosisio Parini, LC, Italy; 3 Molecular Biology Lab, Scientific Institute, IRCCS Eugenio Medea, Bosisio Parini, LC, Italy; 4 Neuroradiology Unit, Fondazione IRCCS Ca' Granda Ospedale Maggiore Policlinico, Milano, Italy; 5 Department of Neurosciences and Mental Health, Fondazione IRCCS Ca' Granda Ospedale Maggiore Policlinico, University of Milan, Milano, Italy; 6 Bioinformatics Lab, Scientific Institute, IRCCS Eugenio Medea, Bosisio Parini, LC, Italy; 7 Department of Psychiatry and Behavioral Neurosciences, University of Texas at Houston, Houston, TX, United States of America; 8 Neuropsychiatry and Neurorehabilitation Unit, Scientific Institute, IRCCS Eugenio Medea, Bosisio Parini, LC, Italy; Hopital Robert Debre, FRANCE

## Abstract

Very preterm (VPT) infants admitted to Neonatal Intensive Care Unit (NICU) are at risk for altered brain growth and less-than-optimal socio-emotional development. Recent research suggests that early NICU-related stress contributes to socio-emotional impairments in VPT infants at 3 months through epigenetic regulation (i.e., DNA methylation) of the serotonin transporter gene (*SLC6A4*). In the present longitudinal study we assessed: (a) the effects of NICU-related stress and *SLC6A4* methylation variations from birth to discharge on brain development at term equivalent age (TEA); (b) the association between brain volume at TEA and socio-emotional development (i.e., Personal-Social scale of Griffith Mental Development Scales, GMDS) at 12 months corrected age (CA). Twenty-four infants had complete data at 12-month-age. *SLC6A4* methylation was measured at a specific CpG previously associated with NICU-related stress and socio-emotional stress. Findings confirmed that higher NICU-related stress associated with greater increase of *SLC6A4* methylation at NICU discharge. Moreover, higher *SLC6A4* discharge methylation was associated with reduced anterior temporal lobe (ATL) volume at TEA, which in turn was significantly associated with less-than-optimal GMDS Personal-Social scale score at 12 months CA. The reduced ATL volume at TEA mediated the pathway linking stress-related increase in *SLC6A4* methylation at NICU discharge and socio-emotional development at 12 months CA. These findings suggest that early adversity-related epigenetic changes might contribute to the long-lasting programming of socio-emotional development in VPT infants through epigenetic regulation and structural modifications of the developing brain.

## Introduction

Even in the absence of severe comorbidities, very preterm (VPT) infants (gestational age at birth < 32 weeks) need long-lasting hospitalization in the Neonatal Intensive Care Units (NICU) [1] and are at risk for altered socio-emotional development [2]. During NICU stay, VPT infants are exposed to life-saving yet invasive interventions including mechanical ventilation and painful skin-breaking procedures. These sources of NICU-related stress have been found to contribute to VPTs’ socio-emotional development during infancy [3, 4] and childhood [5, 6]. Both functional (e.g., epigenetic mechanisms [7]) and structural (e.g., brain volume alterations [8]) factors have been suggested to be involved in setting the risk of less-than-optimal socio-emotional development in VPT infants and children. Nonetheless, the specific mechanisms implicated in the effects of NICU-related stress on socio-emotional developmental outcomes are unknown. Here, using data from a longitudinal research study, we examined potential links between early NICU stress exposure and socio-emotional development at 12 months corrected age (CA) in VPT children, assessing both epigenetic variations (i.e., serotonin transporter gene (*SLC6A4*) methylation [9]) and brain growth (i.e., anterior temporal lobe volume [10, 11]).

Preterm infants’ developing brain is vulnerable to adverse environmental stimulations and advanced Magnetic Resonance Imaging (MRI) techniques have been used to investigate the pathophysiological basis of neurodevelopmental disorders that VPT infants may manifest later in childhood [12]. NICU-related stress might affect VPT infants’ cerebral growth [8, 13]. Previous studies suggested that the anterior temporal lobe (ATL) plays a critical role in socio-emotional functioning and emotion regulation [14, 15] and preterm infants present reduced ATL volume at term age compared to their full-term counterparts [16]. Moreover, from the anatomical point of view, the ATL is of particular interest since it contains the amygdala, extended amygdala and anterior hippocampus, anatomic brain structures that are well-known for their involvement in socio-emotional development and functioning [17, 18]. In the light of this evidence, the ATL is a candidate region of interest (ROI) to examine preterm infants brain growth, consistent with the aims of the present study.

Recent research suggests that early life adversities may contribute to the long-lasting programming of socio-emotional development through functional modifications (e.g., DNA methylation) of stress-related genes, without structural modifications of the chromatin structure [19]. DNA methylation consists in the addition of a methyl group to cytosine/guanine DNA dinucleotides (i.e., CpG sites) within the promoter region of a specific gene usually resulting in reduced transcriptional activity (i.e., gene silencing) [20]. The *SLC6A4* gene codes for the serotonin transporter, it is susceptible to epigenetic regulation by DNA methylation [21] and it acts as the key regulator of the serotonergic system under stress [22]. Greater exposure to NICU-related stress has been recently associated with increased CpG-specific methylation of the *SLC6A4* gene in VPT infants [9], which in turn was predictive of socio-emotional development at 3 months CA [23].

**Fig 1:**
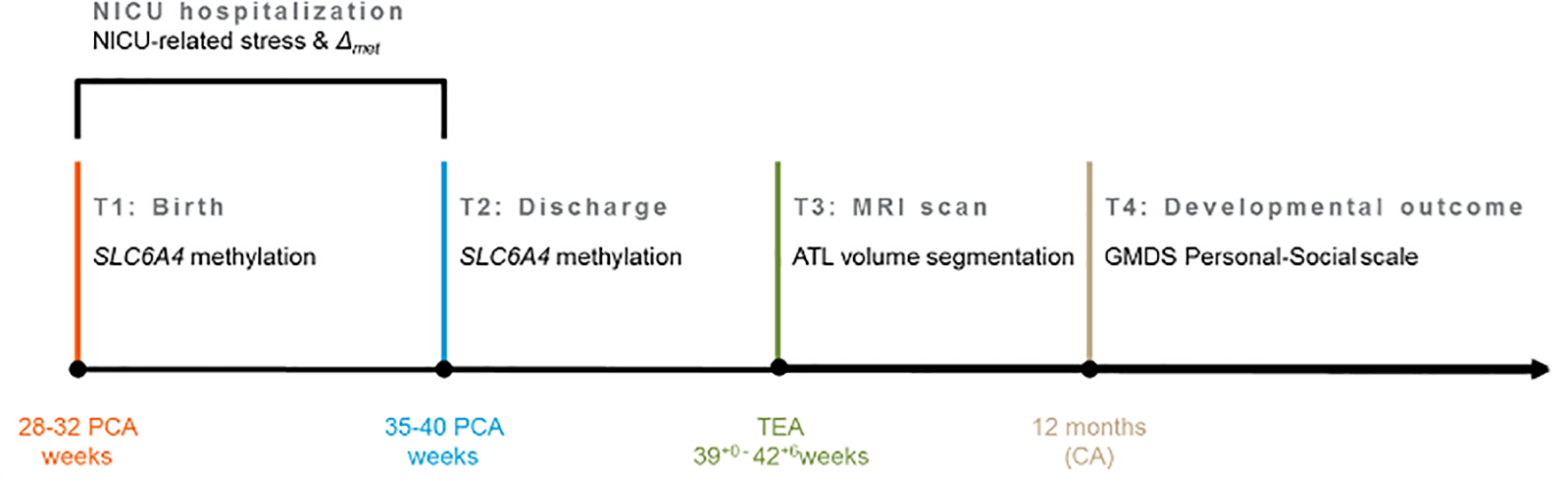
Schematic time-line of the longitudinal project, limitedly to the variables of interest. Note. NICU, Neonatal Intensive Care Unit; *SLC6A4*, serotonin transporter gene; *Δ*_*met*_, mean change in *SLC6A4* methylation from birth to NICU discharge at CpG chr17: 28562786–28562787; ATL, anterior temporal lobe; MRI, Magnetic Resonance Imaging; GMDS, Griffith Mental Development Scales; PCA, Post-Conceptional Age; TEA, term-equivalent age; CA, Corrected Age for prematurity.

Our group has previously documented the epigenetic effects of early pain-related stress exposure on the socio-emotional developmental outcomes of VPT infants during the first months of life [9, 23]. In the present study, we extended previous research assessing the potential links between *SLC6A4* CpG-specific methylation and brain volumes at term equivalent age (TEA). Furthermore, we assessed whether functional (i.e., DNA methylation) and structural brain variations (i.e., ATL volume) might be associated with socio-emotional development at 12 months CA (see Fig 1). The main aims were: (1) to assess the association between increased *SLC6A4* methylation at NICU discharge and ATL volume at TEA; (2) to investigate the effects of altered *SLC6A4* methylation at discharge and ATL volume at TEA on VPT infants performance at the Griffith Mental Development Scales (GMDS [24]) Personal-Social scale at 12 months CA. Finally, through a path analysis, we speculatively tested the hypothesis that an altered ATL volume might mediate the association between early NICU-related *SLC6A4* epigenetic alterations (i.e., increased CpG methylation) and socio-emotional development at 12 months CA.

## Materials and methods

### Participants

Fifty-six VPT infants (<32 weeks of gestation and/or <1500 g at birth) were recruited between October 2011 and April 2014, at the NICU of the Department of Clinical Sciences and Community Health, Fondazione IRCCS Ca’ Granda Ospedale Maggiore Policlinico of Milan. Exclusion criteria included: mothers with documented cognitive impairment and under psychotropic treatment during and after pregnancy; any kind of hemodynamic disturbances experienced during NICU stay (defined as need of inotropic drugs to maintain normal values of arterial blood pressure); need of surgery; major brain lesions as documented by cerebral ultrasound (intraventricular hemorrhage > grade 2 according to Papile [25], cystic periventricular leukomalacia); neuro-sensorial deficits (retinopathy of prematurity ≥ stage 2 [26]); genetic syndromes and/or major malformations. All mothers were Italian, 18-year-old or more, cohabitant with the father of the infant.

### Procedures

The research was conducted in accordance with the Declaration of the World Medical Association and with the 7^th^ revision of the Declaration of Helsinki for ethical principles regarding human experimentation. The study protocol was approved by the ethical committees of the Scientific Institute IRCCS E. Medea in Bosisio Parini and of the Fondazione IRCCS Ca’ Granda Ospedale Maggiore Policlinico of Milan. All parents signed a written informed consent form. For the purposes of epigenetic analyses, cord blood at birth and peripheral blood at NICU discharge were collected from the included VPT infants. Blood samples were obtained by trained nurses to avoid hemolysis and immediately stored at -20°C. Infants’ clinical variables were obtained from medical records at the end of the NICU hospitalization. Mothers completed a socio-demographic form during the first days after birth. Among the initial sample of 56 VPT infants, 12 babies did not undergo brain magnetic resonance imaging (MRI), 11 were scanned after 43 weeks post-menstrual age and 6 MRI scans were not suitable for volumetric analysis due to movement artifacts. As such, 27 subjects (48%) had available MRI at TEA (39^+0^–42^+6^weeks). At 12 months (corrected age for prematurity), developmental functioning was assessed with the GMDS. Three infants (11%) had no GMDS assessment at 12 months.

### Measures

#### Neonatal and clinical variables

Gestational age (weeks) and weight (grams) were recorded at birth. Other neonatal variables included: gender, Apgar at minute 1, twin pregnancy, mode of delivery, being small for gestational age. NICU stress-related variables included: total length of hospitalization (days); number of skin-breaking procedures (e.g., heel lance, arterial and venous punctures, peripheral venous line insertion); total days of invasive ventilation. Other clinical variables included: sepsis, bronchopulmonary dysplasia, necrotizing enterocolitis, retinopathy of prematurity. The Clinical Risk Index for Babies (CRIB II [27]) score was computed to obtain an overall assessment of the clinical risk associated with preterm birth.

#### Socio-demographic variables

Socio-demographic data (i.e., maternal age, years of study and occupation) were obtained from all the mothers. Hollingshead’s classification [28] was used to assess maternal socio-economic status (SES). SES ranged from 0 (occupations that do not require a high school degree) to 90 (occupations that require highly specialized education and training).

#### SLC6A4 methylation

Consistent with previous research [9, 29], we analyzed a CpG-rich region of the *SLC6A4* promoter (chr17:28562750–28562958, Human hg19 Assembly), between -69 and -213 relative to the transcriptional start site, which contains 20 CpG sites and is adjacent to exon 1A (see S1 Table for CpG positions). DNA methylation levels were determined using bisulfite modification followed by PCR amplification and followed by Next-Generation Sequencing (NGS). Genomic DNA was extracted from 0.2 ml of each sample using a GenElute Blood Genomic DNA kit (Sigma). Bisulfite conversion was performed on 500 ng of genomic DNA using the EZ DNA methylation kit (ZymoResearch, Inc., Irvine, CA, USA). Primers were designed using Bisulfite Primer Seeker. A TruSeq amplicon-specific tail 5′ CCTACACGACGCTCTTCCGATCT 3′ was added to the forward primer, while the sequence 5′ TCAGACGTGTGCTCAACCGATCT 3′ was added to the reverse primer, in order to allow synthesis and sequencing of TruSeq libraries of methylated fragments. Primary PCR-amplification was performed on 20 ng of bisulfite- treated DNA using Taq Gold (Life Technologies, Inc.). Cycling comprised 5 min pre-activation at 95°C, followed by 35 cycles of 94°C denaturation for 15 s, 58°C annealing for 20 s, 72°C elongation for 1.5 min. All PCR products were verified on a 2% agarose gel and treated with Ilustra Exo Pro-STAR (GE Healthcare) to eliminate unincorporated primers. Secondary PCR was conducted on each sample using a TruSeq Custom Amplicon Index Kit (Illumina) containing eight forward (i5) and twelve reverse (i7) index primers. Optimal annealing temperature (68°C) and number of PCR cycles (16) were experimentally determined. Cycling comprised 5 min pre-activation at 95°C, followed by 16 cycles of 94°C denaturation for 15 s, 68°C annealing for 20 s, 72°C elongation for 1 min. All PCR products were checked on 2% agarose gel, and approximately equimolar aliquots of each product were pooled and purified on a 2% agarose gel. The purified library was quantified on a Bioanalyzer 2100 (Agilent) and sequenced on a MiSeq (Illumina) using a v2 Reagent kit, 300 cycles PE. Paired-end reads from each sample were independently aligned to the reference sequence by a parallel striped Smith-Waterman algorithm. Only paired reads that aligned coherently to the reference sequence were retained. At each CpG site, the four base-frequencies were evaluated and reported along with the C-to-T percentage.

#### Brain volumes

Brain MRI was performed at TEA on a 3T Philips Achieva scanner (Philips Medical Systems, Best, The Netherlands) according to the internal scanning protocol, reported in Table 1. Patients were fed and wrapped, noise attenuators (MiniMuffs® Natus Medical Inc, San Carlos, California) were applied for hearing protection; heart rate and arterial oxygen saturation were continuously monitored and a neonatologist was present throughout the entire examination. All babies were scanned during spontaneous sleep.

**Table 1:** Scanning protocol for included infants.

Sequences	Slice orientation / thickness	FOV	TR / TE	Matrix
T1 3D FFE	Coronal/ voxel size: 0.9x0.9x0.9 mm	130x140	8.5 / 4.6	144 x 132
T2 TSE	Axial / 2 mm	130x130	7000 / 140	324 x 260
T2 TSE	Coronal / 2 mm	130x130	9000 / 150	324 x 260
T2 FFE	Axial / 3 mm	160x160	652/16	159 x 200
DWI	Axial / 3 mm	180x180	4388 / 55	148 x 127

Note. FOV, Field of View; TR/TE, repetition time / echo time; FEE; Fast Field Echo, TSE, Turbo Spin Echo; DWI, Diffusion Weight Imaging.

#### Socio-emotional development

The GMDS [24] assesses mental development of infants from birth to 8 years. It is based on five subscales tapping the following domains: locomotor, personal-social development, hearing and speech, hand and eye coordination, and performance. For the aims of the current study we used the Personal-Social dimension of the GMDS. This scale assesses the children proficiency in daily living activities, levels of independence, as well as ability to interact with other children. The evaluation of this dimension at 12 months includes items assessing specific competences of personal-social functioning such as visual recognizing of maternal face, following moving persons with eyes, social smiling and vocalizations, self-soothing capacities, smiling and playing in response to mirror image, playing actively in interactive games with others, being interested in activities of others. A final standardized score (ranging from 50 to 150) is obtained, with mean equal to 100 and standard deviation equal to 15. Notably, previous research has documented that this scale is particularly sensitive in capturing early developmental difficulties in VPT infants [30].

### Data reduction

#### NICU-related stress

In order to provide a global measure of NICU-related stress, a Principal Component Analyses was performed on the selected indexes reported above *(i*.*e*., *number of skin-breaking procedures including heel lance*, *arterial and venous punctures*, *peripheral venous line insertion; total days of invasive ventilation)*, leading to a one factor-solution that explained 74% of the variance with factor loadings ranging from .66 to .94. After extracting the principal component (i.e., NICU-related stress) it has been weighted on length of NICU stay (days), in order to obtain a global index of stress exposure in NICU and to avoid confounding effects of multicollinearity in further analyses.

#### SLC6A4 methylation

Previous research documented that CpG-specific (i.e., chr17: 28562786–28562787) *SLC6A4* methylation occurs in VPT infants in response to NICU-related stress [9] and is linked with further socio-emotional development at 3 months CA [23]. This CpG site showed the greatest association with early exposure to NICU-related stress and pain in VPT infants. As such, it could be a possible CpG site candidate for potential epigenetic effects of the adverse NICU environment on VPT infants’ methylation status of the SLC6A4 gene. Consistently, in the present study, we adopted a CpG-specific approach, focusing on the delta-score obtained subtracting *SLC6A4* methylation at birth from *SLC6A4* methylation at NICU discharge for the CpG chr17: 28562786–28562787 (i.e., *Δ*_*met*_*)*.

#### Brain segmentation and volumetric analysis

**Fig 2:**
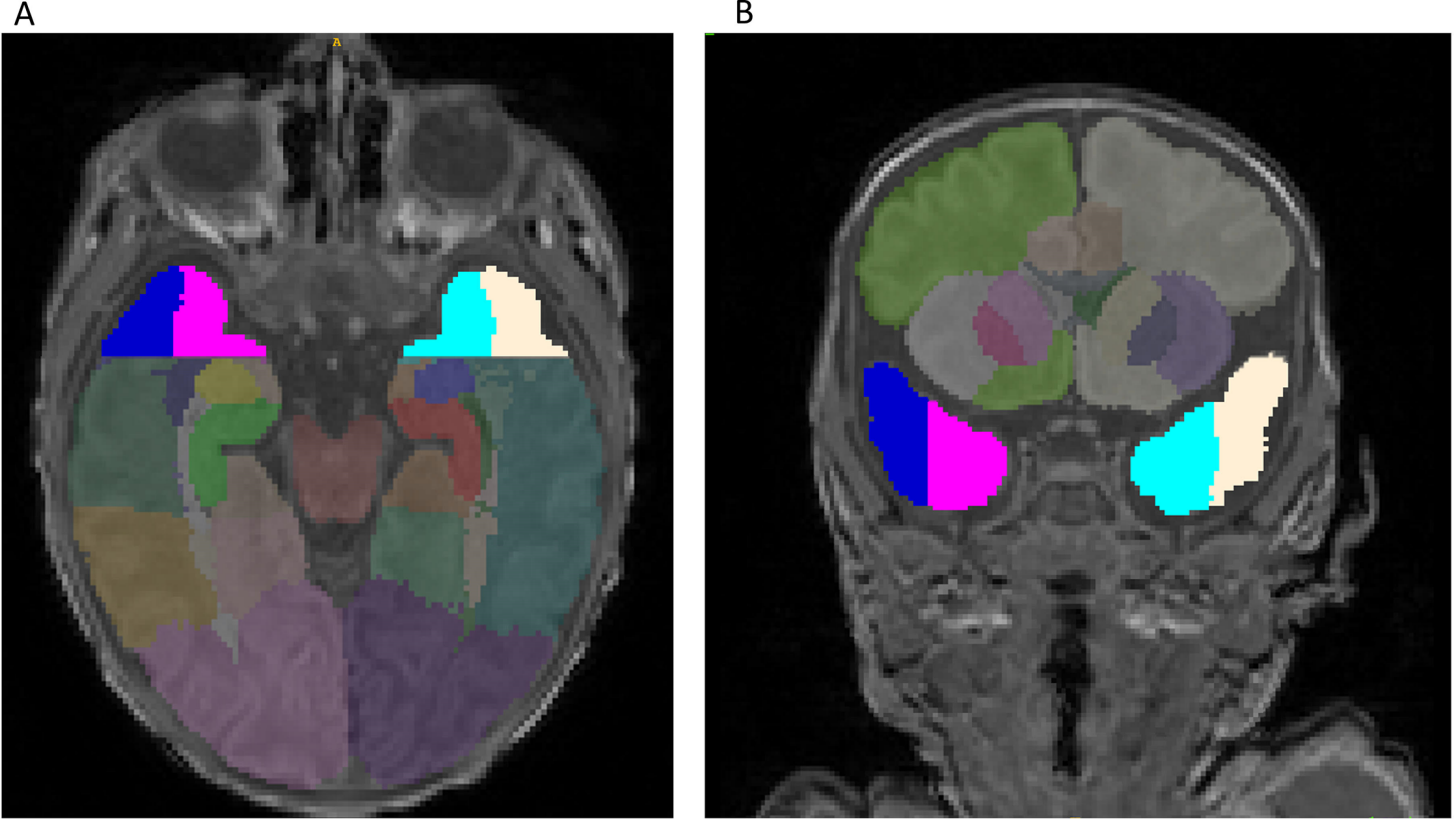
**Brain MRI segmentation: A. axial and B. coronal view T1 images.** Note. Colors highlight anterior temporal lobe (ATL) lateral part left (ATL-LPL, yellow) and right (ATL-LPR, dark blue) as well as ATL medial part left (ATL-M;PL, light blue) and right (ATL-MPR, pink).

Automated segmentation was conducted on each neonatal Axial T2 2 mm scan, in conjunction with the T1 scan. The two images were registered, in order to segment brain tissue and extract volume measures using a neonatal specific segmentation approach [31] based on the Expectation–Maximisation (EM) technique [32]. The target areas were visually checked and manual editing was performed with ITK-SNAP [33]. Volumetric measures of the structures of each neonate were extracted from each segmentation. All measures are expressed in mm^3^. For the purposes of the present study, the following ROIs were selected: anterior temporal lobe lateral part left (ATL-LPL) and right (ATL-LPR) and anterior temporal lobe medial part left (ATL-MPL) and right (ATL-MPR) (Fig 2).

### Plan of analysis

Descriptive statistics for neonatal, clinical and socio-demographic characteristics as well as for variables of interest (i.e., NICU-related stress, *Δmet*, ATL volumes, socio-emotional development) have been computed. The regression of NICU-related stress on *Δmet* has been carried to confirm previous findings on a larger sample from the same longitudinal study.

#### Effects of NICU-related stress and SLC6A4 methylation on ATL brain volumes

In the first step, we performed different multiple regressions to test the effects of NICU-related stress and *SLC6A4* methylation (i.e., *Δmet*) on each ROI, controlling for the following covariates: gestational age at birth, maternal SES and gestational age at MRI.

#### Effects of brain volumes on developmental outcome

In the second step, we performed a series of multiple regressions to predict the performance on the Griffith subscale Personal-Social. In order to reduce the number of predictors in the face of limited sample size, we weighted brain volumes for age at the MRI, extracting the residuals of the regression of gestational age at MRI on brain volumes. We added the extracted weighted measures (i.e., ATL-LPL_w_; ATL-LPR_w_; ATL-MPL_w_; ATL-MPR_w_) in separate regression models together with NICU-related stress, gestational age at birth and maternal SES.

#### Exploratory assessment of the brain volume mediation hypothesis

Finally, we performed four different path analyses, one for each ROI. Specifically, we tested the indirect effect of methylation on the Griffiths’ Personal-Social scale, mediated by brain volumes. The goodness of the model was assessed separately for each ROI according to: non-significant chi-square statistic; comparative fit index (CFI) and Tucker-Lewis index (TLI) close to .95 [34]; Root Mean Squared Error of Approximation (RMSEA) smaller than .05; Square Residual Root Mean (SRMR) smaller than .08 [35]; non-significant *p* value associated with the CI of RMSEA [36].

## Results

Descriptive statistics for socio-demographic characteristics and variables of interest are reported in Table 2. The association between NICU-related stress and *Δmet* from birth to discharge was positive and significant (*β* = .43, *p* = .02, 95% C. I. [.06,.80]).

**Table 2:** Descriptive statistics for the included subjects.

**Infants' characteristics**	**Mean**	**SD**
Gestational age at birth (weeks)	30.10	2.00
Birth weight (grams)	1285.00	277.00
CRIB II score *	5.80	2.70
Days of ventilation	19.20	17.00
Number of skin-breaking procedures	30.80	19.00
Length of NICU stay (days)	52.80	22.90
Gestational age at MRI scan (weeks)	40.80	0.90
	**N**	**%**
Males	12.00	44.00
Singleton	12.00	44.00
Cesarean Section	27.00	100.00
Assisted ventilation	26.00	96.20
Small for gestational age ^†^	3.00	11.00
Sepsis ^‡^	5.00	18.50
Bronchopulmonary dysplasia [37]	1.00	3.70
Necrotizing enterocolitis [38]	2.00	7.40
Retinopathy of prematurity ^§^	2.00	7.40
**Maternal characteristics**	**Mean**	**SD**
Age (years)	35.17	4.62
Education (years of study)	16.59	2.15
Socio-economic status (SES) ^¶^	51.48	19.16
**Variables of interest**	**Mean**	**SD**
NICU-related stress	0.00	1.00
*Δmet* ^#^	0.07	1.23
ATL-MPR	1674.31	309.91
ATL-MPL	1758.76	366.96
ATL-LPR	1957.52	306.05
ATL-LPL	1937.60	321.38
GMDS personal-social scale score	94.88	9.60

Note.

* Clinical Risk Index for Babies–II [27]

^†^ Small for gestational age is defined as birth weight inferior to the 10th centile

^‡^ Sepsis is defined as clinical signs of infection (including tachycardia/bradycardia, hypotension, poor perfusion, apnoea, cyanosis, tachypnea, need for ventilator, increased oxygen requirement, abnormal temperature, lethargy, hypotonia and feeding problems) associated with increased plasmatic levels of C reactive protein and a positive blood colture

^§^ Stage I retinopathy of prematurity, according to the International Classification [26]

^¶^ According to Hollingshead classification [28]

^#^ *Δ*_*met*_ *SLC6A4* methylation at NICU discharge *minus SLC6A4* methylation at birth at CpG chr17: 28562786–28562787; ATL-MPR, anterior temporal lobe–medial part right; ATL-MPL, anterior temporal lobe–medial part left; ATL-LPR, anterior temporal lobe–lateral part right; ATL-LPL, anterior temporal lobe–lateral part left; GMDS, Griffiths Mental Development Scales [24].

### Effects of NICU-related stress and SLC6A4 methylation on brain volumes

Results of the first step of analysis are presented in Table 3. A significant effect of *Δmet* on ROI volumes emerged for all the ATL areas investigated (ATL-MPR, *F*(5,21) = 2.52, *p* = .06, *R*^*2*^ = 0.37; ATL-MPL, *F*(5,21) = 2.84, *p* = .04, *R*^*2*^ = 0.40; ATL-LPR, *F*(5,21) = 2.52, *p* = .07, *R*^*2*^ = 0.36; ATL-LPL, *F*(5,21) = 2.60, *p* = .05, *R*^*2*^ = 0.38). Greater increase in methylation was associated with smaller brain volume in the ROI (see Table 3).

**Table 3:** Effects of NICU-related stress and SLC6A4 methylation on brain volumes.

Predictors	Outcome Variables							
	Anterior Temporal Lobe—Medial Part							
	**ATL-MPR**				**ATL-MPL**			
	*B*	SE	*t*	*p*	*B*	SE	*t*	*p*
Gestational age at birth	.00	.15	-.03	.98	-.10	.15	-.66	.51
NICU-related stress	-.06	.31	-.20	.84	-.08	.30	-.26	.80
*Δmet*	-.48	.16	-3.06	.01	-.54	.15	-3.50	.00
SES	.01	.01	.63	.54	.01	.01	.92	.37
Gestational age at MRI	.11	.21	.54	.59	.24	.20	1.16	.26
	**ATL-LPR**				**ATL-LPL**			
	*B*	SE	*t*	*p*	*B*	SE	*t*	*p*
Gestational age at birth	-.17	.15	-1.15	.26	-.28	.15	-1.85	.08
NICU-related stress	-.20	.31	-.64	.53	-.33	.31	-1.07	.30
*Δmet*	-.44	.16	-2.76	.01	-.37	.16	-2.32	.03
SES	.01	.01	.73	.47	.01	.01	1.00	.33
Gestational age at MRI	.43	.21	2.02	.06	.53	.21	2.55	.02

Note. ATL-MPR, anterior temporal lobe–medial part right; ATL-MPL, anterior temporal lobe–medial part left; ATL-LPR, anterior temporal lobe–lateral part right; ATL-LPL, anterior temporal lobe–lateral part left; NICU, Neonatal Intensive Care Unit; *Δmet*, mean change in *SLC6A4* methylation from birth to NICU discharge at CpG chr17: 28562786–28562787; SES, socio-economic status; MRI, magnetic resonance imaging.

### Effects of brain volumes on socio-emotional development

Significant effects emerged for ATL-MPL_w_, *R*^*2*^ = .58, *F*(5,18) = 5.00, *p* = .005, ATL-LPR_w_, *R*^*2*^ = .61, *F*(5,18) = 5.62, *p* = .003, and ATL-LPL_w_, *R*^*2*^ = .68, *F*(5,18) = 7.61, *p* = .0005,. A significant effect of ATL volumes on the GMDS Personal-Social scale emerged for ATL-MPL_w_, ATL-LPR_w_ and ATL-LPL_w_ (Table 4). The greater these areas were, the higher was the score of the GMDS Personal-Social scale. In addition, higher scores in the GMDS Personal-Social scale were significantly predicted by lower NICU-related stress and gestational age at birth.

**Table 4:** Effects of brain volumes on GMDS Personal-Social scale.

	Personal-Social scale					Personal-Social scale			
**Predictors**	*B*	SE	*t*	*p*		*B*	SE	*t*	*p*
Gestational age at birth	-0.67	0.19	-3.62	0.00	Gestational age at birth	-0.63	0.16	-3.83	0.00
NICU-related stress	-1.40	0.36	-3.89	0.00	NICU-related stress	-1.37	0.32	-4.27	0.00
*Δmet*	0.04	0.18	0.24	0.81	*Δmet*	0.17	0.17	1.01	0.33
ATL-MPR_w_	0.00	0.00	1.31	0.21	ATL-MPL_w_	0.01	0.00	2.57	0.02
SES	0.00	0.01	0.45	0.66	SES	0.00	0.01	0.25	0.81
**Predictors**	*B*	SE	*t*	*p*	** **	*B*	SE	*t*	*p*
Gestational age at birth	-0.59	0.16	-3.70	0.00	Gestational age at birth	-0.47	0.15	-3.12	0.01
NICU-related stress	-1.31	0.31	-4.23	0.00	NICU-related stress	-1.13	0.29	-3.95	0.00
*Δmet*	0.14	0.15	0.92	0.37	*Δmet*	0.15	0.13	1.11	0.28
ATL-LPR_w_	0.01	0.00	2.89	0.01	ATL-LPL_w_	0.01	0.00	3.75	0.00
SES	0.00	0.01	0.29	0.78	SES	0.00	0.01	-0.10	0.93

Note. ATL-MPR_w_, anterior temporal lobe–medial part right weighted on gestational age at MRI; ATL-MPL_w_, anterior temporal lobe–medial part left weighted on gestational age at MRI; ATL-LPR_w_, anterior temporal lobe–lateral part right weighted on gestational age at MRI; ATL-LPL_w_, anterior temporal lobe–lateral part left weighted on gestational age at MRI; NICU, Neonatal Intensive Care Unit; *Δmet*, mean change in *SLC6A4* methylation from birth to NICU discharge at CpG chr17: 28562786–28562787; SES, socio-economic status; MRI, magnetic resonance imaging.

### Exploratory assessment of the brain volume mediation hypothesis

The path analysis is reported in S1 Fig. All the indexes indicated an acceptable fit of the data: all chi-squares < 0.7, *p* > .6;; CFIs = 1.00; TLIs > 1.00; RMSEAs = 0.00, *ps* > .69. The mediated path was significant for three out of four ROI analyzed (i.e., ATL-MPL, ATL-LPR, ATL-LPL). More specifically, the analysis suggested that (1) higher NICU-related stress was significantly associated with greater *Δmet*; (2) greater *Δmet* was significantly associated with reduced ATL volumes at TEA; (3) reduced ATL volumes were significantly associated with lower scores at the GMDS Personal-Social scale at 12 months CA. Finally, NICU-related remained a significant predictor of GMDS Personal-Social score at 12 months CA.

## Discussion

In this prospective longitudinal study, we tested the effects of NICU-related stress, CpG-specific *SLC6A4* methylation and ATL volumes on socio-emotional development of VPT infants at 12 months CA. First, we tested NICU-related stress and *SLC6A4* CpG-specific methylation in association with brain growth at TEA in VPT infants. After controlling for potential perinatal confounders, CpG-specific *SLC6A4* methylation emerged as a significant predictor of bilateral ATL volume, including both medial and lateral areas. In previous studies, we had shown that the birth-to-discharge methylation increase of this specific CpG site within the promoter region of the serotonin transporter gene was affected by early exposure to skin-breaking procedures during the NICU hospitalization in VPT infants [9]. Moreover, higher methylation of this CpG site was predictive of poorer socio-emotional regulation at 3 months CA, when VPT infants were compared with full-term peers [23]. As such, the present findings extend previous evidence suggesting that early NICU-related stress might also be associated with reduced bilateral ATL volume at TEA in VPT infants, via epigenetic regulation (i.e., increased promoter region methylation) of the serotonin transporter gene.

Second, we documented that reduced ATL volumes were significantly associated with lower scores on the Personal-Social scale of the GMDS at 12 months CA. This finding corroborates previous research [39, 40] that already suggested that the ATL is involved in socio-emotional functioning [14, 15]. Notably, the GMDS Personal-Social scale includes the assessment of infants’ face recognition, adequate response to social stimuli and self-regulation, which are specific socio-emotional processes associated with ATL activation. It should be noted that the scores in the Personal-Social scale observed at 12 months CA were within normal range (min 81; max 103) and they did not exceed one standard deviation from the mean expected value. Also, VPT infants enrolled in this study did not develop severe comorbidities related to preterm birth and had no major brain lesions at conventional MRI. Thus, it seems that early NICU-related stress could, at least partially, explain the emergence of individual variability in socio-emotional development at 12 months CA in VPT infants.

Notably, at an exploratory level, ATL volumes emerged as significant mediators of the association between *SLC6A4* CpG-specific methylation and Personal-Social score at 12 months CA. Given the well-recognized role played by the serotonergic system in socio-emotional regulation and development [22], this finding is intriguing. A speculative interpretation might be that the early epigenetic variations (i.e., *SLC6A4* methylation) observed in association with exposure to life adversities (i.e., NICU-related stress) might be embedded in the developing biology of young at-risk individuals (i.e., VPT infants) through cerebral developmental changes (i.e., ATL volume reduction), finally leading to long-term (i.e., 12 months CA) reduced socio-emotional capacities. Future research is warranted to corroborate the potential pathway through which early NICU-related epigenetic variations might contribute to the long-term socio-emotional development of VPT infants by modifications of specific brain structures.

This study has limitations. First, the sample size was relatively small. As such, limited control for confounders was possible and generalizability is not warranted. Second, the analyses were performed on different tissues at birth (i.e., cord blood) and discharge (i.e., peripheral blood). Tissue specificity and the relevance of non-central tissues in human behavioral epigenetics research is discussed in literature [41]. Nonetheless, it should be highlighted that cord blood and peripheral blood DNA are both commonplace in epidemiological studies [42]. Moreover, cord blood and peripheral blood samples have been suggested to reveal similar levels of DNA methylation in humans [43]. Additionally, we did not perform any immunologic analysis to ascertain the white blood cell distribution in our peripheral and cord blood samples; therefore we are unable to correct our results for cell content. Third, the number of skin-breaking procedures has been collected retrospectively and only effective blood sampling and venous line insertions have been recorded. Attempts were not recorded thus resulting in possible underestimation of the number of skin-breaking procedures the infants underwent during NICU stay. We cannot rule out that other early NICU stressors (i.e., maternal separation) might have contributed to our findings. Fourth, genetic variants of the *SLC6A4* gene (e.g., 5-HTTLPR) are known to be involved in the availability of serotonin transporter [44], in socio-emotional development [45, 46] and they might interact with epigenetic mechanisms in human beings [14]. We suggest that future research is needed to assess the effects of early epigenetic variations of *SLC6A4* on VPT infants’ brain development in the context of different 5-HTTLPR genotypes. Fifth, a single-CpG approach has been adopted in the present study. This approach was based on the fact the sample size was relatively small and multiple comparisons using all the 20 CpG sites would have resulted in critically underpowered statistics. Moreover, the choice of the specific CpG site of the SLC6A4 studied here was based on previous research suggesting that this site shows the highest sensitivity to environmental stress in preterm infants among the pool of 20 CpG sites investigated by our group [47]. Future studies on bigger samples are warranted to look at the association between methylation assessed at different CpG sites and brain volume measures in this population. Finally, in the light of the limited sample size, the mediation-testing path analysis must be intended as an exploratory appreciation of the complex relationships occurring between environmental, epigenetic, cerebral and behavioral variables.

In conclusion, the present study suggests the intriguing hypothesis that the effects of early NICU-related stress on VPT infants’ socio-emotional development at 12 months CA might be affected and at least partially mediated by both functional mechanisms (i.e., increased *SLC6A4* methylation at discharge) and structural changes in the developing brain (i.e., reduced ATL volumes at TEA). This is consistent with emerging hypotheses about the brain effects of early adversity-related epigenetic alterations in humans [48] and the present findings have implications for both research and clinical practice.

First, these findings further expand our knowledge of the role that early NICU-related stress might play in affecting brain growth and development in VPT infants. Indeed, previous work have documented that formerly preterm infants and children might have altered brain development in specific areas [49]. Moreover, exposure to pain and invasive procedures during the NICU stay might have a long-standing impact on brain architecture during infancy and childhood [50]. The present work preliminarily suggests that epigenetics variations (i.e., methylation) might be a potential mechanism through which the early exposure to NICU-related adversity might contribute to altered brain development in specific areas, which in turn could be associated with long-term programming of VPT infants’ developmental outcomes even several months after discharge [51]. Future prospective and longitudinal research is warranted to help us understand the complex interplay among early NICU-related adversities, epigenetic variations and brain development in contributing to setting the risk for less-than-optimal socio-emotional development in VPT infants.

As for clinical implications, the present results are particularly intriguing in the context of previous works suggesting that early interventions during the NICU stay might have an impact on the neurodevelopment of preterm infants [52,53]. For example, Als and colleagues [54] have demonstrated that a specific and comprehensive program of developmental care in the NICU (i.e., the Newborn Individualized Developmental Care and Assessment Program, NIDCAP) is associated with increased coherence between frontal and occipital brain regions, higher anisotropy in left internal capsule with a trend for frontal white matter. More recently, a randomized controlled trial on the effects of an early training of parental sensitivity during the NICU stay demonstrated that both maturation and connectivity of white matter of VPT infants were significantly improved in the intervention group compared to controls who did not receive the same parental intervention [55]. In the light of this evidence, one might wonder whether early NICU interventions involving parents of VPT infants might have long-lasting impact on infants’ brain development through epigenetic mechanisms. Consistently, the study of the epigenetic correlates of developmental care effects on VPT infants’ neurobehavioral, cognitive and emotional development appear to be a promising direction of future research in the context of Preterm Behavioral Epigenetics [47, 56].

## Supporting information

S1 Fig **Exploratory path analysis for the mediation effect of ATL-MPL (A), ATL-LPR (B), and ATL-LPL (C) on the relationship between birth-to-discharge SLC6A4 methylation increase and GMDS Personal-Social score at 12 months CA.** Note. NICU, Neonatal Intensive Care Unit; Δmet, mean change in SLC6A4 methylation from birth to NICU discharge at CpG chr17: 28562786–28562787; ATL-MPL, anterior temporal lobe–medial part left; ATL-LPR, anterior temporal lobe–lateral part right; ATL-LPL, anterior temporal lobe–lateral part left. Dotted lines represent non-significant associations. Mediated paths: a*b, β = -.31, p = .02, 95% C.I. [-4.51, -.38]; c*d, β = -.28, p = .02, 95% C.I. [-4.00, -.28]; e*f, β = -.29, p = .02, 95% C.I. [-4.18, -.32].
(TIF)

S1 Table CpG sites position on the chromosome 17 and distance from the transcription start site of the SLC6A4 gene. Note. Chr17 = Chromosome 17; TSS = Transcriptional Start Site.
(DOCX)

S1 Dataset This data set reports all relevant data for the present paper. (XLSX)
