# Supplementary figures and images for: From early stress to 12-month development in very preterm infants: Preliminary findings on epigenetic mechanisms and brain growth
Source: PLoS One. 2018 Jan 5;13(1):e0190602. doi: 10.1371/journal.pone.0190602 (PMC5755830; doi:10.1371/journal.pone.0190602)

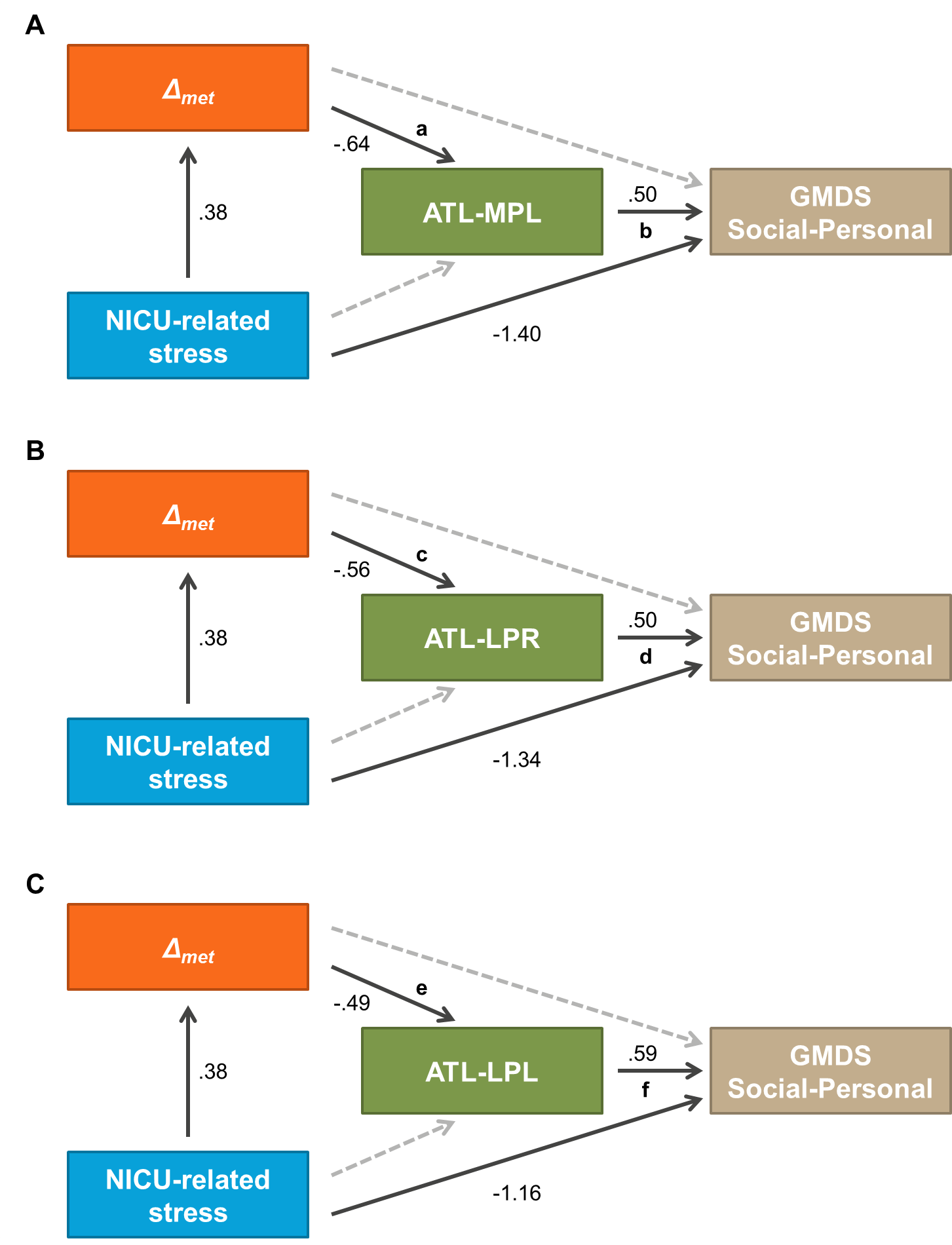

Supplement: S1 Fig — Exploratory path analysis for the mediation effect of ATL-MPL (A), ATL-LPR (B), and ATL-LPL (C) on the relationship between birth-to-discharge SLC6A4 methylation increase and GMDS Personal-Social score at 12 months CA. Note. NICU, Neonatal Intensive Care Unit; Δmet, mean change in SLC6A4 methylation from birth to NICU discharge at CpG chr17: 28562786–28562787; ATL-MPL, anterior temporal lobe–medial part left; ATL-LPR, anterior temporal lobe–lateral part right; ATL-LPL, anterior temporal lobe–lateral part left. Dotted lines represent non-significant associations. Mediated paths: a*b, β = -.31, p = .02, 95% C.I. [-4.51, -.38]; c*d, β = -.28, p = .02, 95% C.I. [-4.00, -.28]; e*f, β = -.29, p = .02, 95% C.I. [-4.18, -.32]. (TIF) [file pone.0190602.s001.tif]
