# Supplementary material for: From early stress to 12-month development in very preterm infants: Preliminary findings on epigenetic mechanisms and brain growth
Source: PLoS One. 2018 Jan 5;13(1):e0190602. doi: 10.1371/journal.pone.0190602 (PMC5755830; doi:10.1371/journal.pone.0190602)
Supplement: S1 Table — Note. Chr17 = Chromosome 17; TSS = Transcriptional Start Site. (DOCX) [file pone.0190602.s002.docx]

**S1 Table. CpG sites position on the chromosome 17 and distance from the transcription start site of the SLC6A4 gene**

| CpG progressive number | Dinucleotide position on chromosome 17 | Distance from the TSS |
| --- | --- | --- |
| 1 | Chr17: 28562783-28562784 | -69 |
| 2 | Chr17: 28562786-28562787 | -72 |
| 3 | Chr17: 28562813-28562814 | -99 |
| 4 | Chr17: 28562826-28562827 | -112 |
| 5 | Chr17: 28562847-28562848 | -133 |
| 6 | Chr17: 28562849-28562850 | -135 |
| 7 | Chr17: 28562853-28562854 | -139 |
| 8 | Chr17: 28562855-28562856 | -141 |
| 9 | Chr17: 28562861-28562862 | -147 |
| 10 | Chr17: 28562863-28562864 | -149 |
| 11 | Chr17: 28562869-28562870 | -155 |
| 12 | Chr17: 28562884-28562855 | -170 |
| 13 | Chr17: 28562888-28562885 | -174 |
| 14 | Chr17: 28562902-28562903 | -188 |
| 15 | Chr17: 28562904-28562905 | -190 |
| 16 | Chr17: 28562909-28562910 | -195 |
| 17 | Chr17: 28562914-28562915 | -200 |
| 18 | Chr17: 28562921-28562922 | -207 |
| 19 | Chr17: 28562923-28562924 | -209 |
| 20 | Chr17: 28562927-28562928 | -213 |

Note. Chr17 = Chromosome 17; TSS = Transcriptional Start Site.
